# Special issue on ultrafine particles: where are they from and how do they affect us?

**DOI:** 10.1038/s12276-020-0395-z

**Published:** 2020-03-17

**Authors:** Goohyeon Hong, Young-Koo Jee

**Affiliations:** Department of Internal Medicine, Dankook University College of Medicine, Cheonan, 31116 Republic of Korea

**Keywords:** Translational research, Respiratory tract diseases

Recently, as air pollution has worsened, social interest in air pollution and its effect on the human body have increased. Air pollution is a mixture of thousands of components that are harmful to human health^1^. Particulate matter (PM) is the principal component of indoor and outdoor air pollution and has been attributed to the most severe health effects^2,3^. PM is a cocktail of chemicals (hydrocarbons, salts and other compounds given off by vehicles, cooking stoves and industry) and other natural components, such as dust and microorganisms^4^. PM includes a range of particle sizes, such as coarse, fine, and ultrafine particles (UFPs).

Particles are usually defined based on their size: coarse particles are those with a diameter of 10 μm or less (PM10), fine particles are those with a diameter of 2.5 μm or less (PM2.5), and UFPs are those with a diameter of 100 nm or less^1,5^. It is known that the increased concentration of PM in the air leads to worsened symptoms of respiratory and cardiovascular disease and increases the prevalence and mortality rate of the disease^1,6,7^. However, most previous studies have been performed on PM10 or PM2.5. There is a growing concern in the public health community about the contribution of UFP to human health. UFPs are classified into two major categories based on their sources. UFP typically refers to a particle that is incidentally generated in the environment, often as a byproduct of fossil fuel combustion, condensation of semivolatile substances or industrial emissions, whereas nanoparticles are manufactured through controlled engineering processes^8^. UFPs can deposit in the distal lung regions (alveoli) and lung parenchyma and penetrate into the heart and bloodstream, which can cause significant damage to the respiratory system, cardiovascular system and all organs.

This special issue, entitled ‘ultrafine particles,’ provides insights into current trending topics in the field of UFPs, including concepts, sources, physicochemical characteristics of UFPs and mechanisms of UFP-induced health effects, moreover the impact of bacteria-derived ultrafine dust particles on pulmonary diseases is covered.

First, the health effects of UFPs are discussed by Schraufnagel^9^. UFPs generally enter the body through the lungs but translocate to essentially all organs. Their toxicity increases as their size decreases and surface area increases and is based on the adsorbed surface material and physical characteristics of the particles. UFPs cause systemic inflammation, endothelial dysfunction, and coagulation changes predisposing patients to ischemic cardiovascular disease and hypertension and are also linked to diabetes and cancer. They can travel up the olfactory nerves to the brain and cause cerebral and autonomic dysfunction. In utero exposure increases the risk of low birth weight. Likewise, UFPs systemically exacerbate and increase the occurrence of various diseases.

The sources and physicochemical characteristics of UFPs are discussed by Kwon et al.^10^. They address the characteristics of UFPs from diesel combustion and elaborate on the role of UFPs in global climate change. The adverse effects of UFPs on meteorological processes and the hydrological cycle may be even more harmful to human health than their direct toxic effects.

Dr. An-Soo Jang’s group^11^ provides a view of the mechanisms of UFP-induced respiratory health effects. Humans can be exposed to UFPs by inhalation. UFPs readily deposit in the airways and centriacinar regions of the lung; they are involved in the pathogenesis of airway inflammation and exacerbate respiratory diseases such as asthma, bronchitis, and chronic obstructive lung disease. The mechanism of UFP-induced human health effects can be explained by oxidative cellular damage associated with innate immunity, adaptive immunity, and reactive oxygen species.

Of note is the impact of biological UFP, principally lipopolysaccharide (LPS) and bacterial extracellular vesicles (EVs), in indoor dust and the pathophysiological mechanisms involved in the development of chronic pulmonary diseases, reviewed by Yang et al.^12^. The impact of LPS-induced pulmonary inflammation is based primarily on the amount of inhaled LPS, which is associated with asthma, COPD, and lung cancer incidence. Responses to bacterial EV exposure can be broadly divided based on one of two mechanisms. Extracellular bacteria-derived EVs can cause neutrophilic inflammation, which is associated with asthma, emphysema, COPD, and lung cancer. In contrast, intracellular bacteria-derived EVs cause mononuclear inflammation, which increases the risk of emphysema. The authors also suggest that the next steps should focus on the overall reduction of LPS sources in addition to the improvement of the balance of inhaled bacterial EVs in the indoor environment to reduce pulmonary disease risk.

These four articles in this special issue provide comprehensive reviews of UFPs and related effects on human health and disorders. Collectively, the articles in this special issue provide an invaluable resource for understanding the current status and future perspectives of UFPs. We hope the review articles presented in this special issue shed light on the significance of UFPs. The potential for UFPs to cause harm to health is great, but their precise role in many illnesses is still unknown; therefore, the focus of air pollution studies should shift to the health effects of UFPs. Global and interdisciplinary research on the components, sources and influence of UFPs on health is urgent.
